# Multi-Walled Carbon Nanotubes Accelerate Leukaemia Development in a Mouse Model

**DOI:** 10.3390/toxics12090646

**Published:** 2024-09-02

**Authors:** Qingqing Wang, Jingdan Han, Mujia Wei, Huikai Miao, Min Zhang, Biao Wu, Yao Chen, Yanwen Zheng, Robert Peter Gale, Bin Yin

**Affiliations:** 1 Clinical Medical Research Center, The Affiliated Wuxi No.2 People’s Hospital of Nanjing Medical University, Wuxi 214002, China; wqq5837@163.com (Q.W.); hjd1356620009@163.com (J.H.); weimujiayouxiang@163.com (M.W.); 2 Department of Laboratory Medicine, Jiangnan University Medical Center, Wuxi 214002, China; miaohuikai1989@163.com (H.M.); mien1030@163.com (M.Z.); wubiao2011@163.com (B.W.); 15861681813@163.com (Y.C.); 3 Cyrus Tang Hematology Center, Jiangsu Institute of Hematology, The First Affiliated Hospital, Soochow University, Suzhou 215123, China; 13914391068@163.com; 4 Haematology Research Centre, Department of Immunology and Inflammation, Imperial College London, London SW7 2AZ, UK; robertpetergale@gmail.com

**Keywords:** multi-walled carbon nanotubes, leukemia, PEG, inflammation, MOL4070LTR

## Abstract

Inflammation is associated with an increased risk of developing various cancers in both animals and humans, primarily solid tumors but also myeloproliferative neoplasms (MPNs), myelodysplastic syndromes (MDS), and acute myeloid leukemia (AML). Multi-walled carbon nanotubes (MWCNTs), a type of carbon nanotubes (CNTs) increasingly used in medical research and other fields, are leading to a rising human exposure. Our study demonstrated that exposing mice to MWCNTs accelerated the progression of spontaneous MOL4070LTR virus-induced leukemia. Additionally, similar exposures elevated pro-inflammatory cytokines such as interleukin (IL)-1β, IL-6, and tumor necrosis factor (TNF)-α and induced reactive oxygen species (ROS) in a murine macrophage cell line. These effects were significantly reduced in immunodeficient mice and when mice were treated with methoxypolyethylene glycol amine (PEG)-modified MWCNTs. These findings underscore the necessity of evaluating the safety of MWCNTs, particularly for those with hematologic cancers.

## 1. Introduction

The applications of nanotechnology are increasingly prevalent in modern society. In the realm of medicine, nanoparticles are being harnessed to deliver drugs more efficiently to targeted cells, thereby enhancing the effectiveness of treatments and minimizing side effects. This precision medicine approach is paving the way for breakthroughs in cancer therapy and the management of chronic diseases [1,2,3,4,5,6,7,8,9,10,11,12,13,14,15,16,17,18,19,20,21,22,23,24,25,26,27,28,29,30,31,32,33,34]. Carbon nanotubes (CNTs) are nanoscale seamless hollow cylinders composed of single- or multi-walled graphene, characterized by unique physical, chemical, and electronic properties. CNTs have been explored in diverse medical applications such as supporting bone and neuron growth, enhancing artificial muscle function, biosensing for biological samples, immune therapy adjuvants, drug delivery systems, optical imaging, and thermo-therapy [5,6,7,8,9,10,11,12,13,14]. More background on the therapeutic uses of MWCNTs can be found in reviews [35,36,37].

The safety of CNTs has been extensively studied in the environmental, occupational, and biomedical contexts [14,15]. Recent evidence suggests that exposure to CNTs can lead to adverse health effects, including genotoxicity [16,17] and the induction of inflammation [18,19,20,21].

Inflammation is associated with an increased risk of various cancers in both animals and humans, predominantly solid tumors but also myeloproliferative neoplasms (MPNs), myelodysplastic syndromes (MDS), and acute myeloid leukemia (AML) [22,23,24,25,26,27,28,29,30].

Multi-walled carbon nanotubes (MWCNTs), a specific form of CNTs increasingly used in medical research and drug formulation, are contributing to increased human exposure. Our study demonstrated that exposing mice to MWCNTs accelerated the development of spontaneous MOL4070LTR virus-induced leukemia. Additionally, similar exposures induced the production of pro-inflammatory cytokines, including IL-1β, IL-6, and TNF-α, and elevated reactive oxygen species (ROS) levels in a murine macrophage cell line. Importantly, these effects were significantly mitigated in immunodeficient mice and in mice treated with methoxypolyethylene glycol amine (PEG)-modified MWCNTs. These findings underscore the necessity of evaluating the safety of drugs formulated with MWCNTs, particularly in those with hematologic cancers.

## 2. Materials and Methods

### 2.1. Preparation of Acidified MWCNTs and PEG-Modified MWCNTs

The methoxypolyethylene glycol amine (PEG) was purchased from Merck KGaA (Darmstadt, Germany, 80506-64-5). H_2_SO_4_ was purchased from Merck KGaA (7664-93-9). HNO_3_ was purchased from Merck KGaA (7697-37-2). N-(3-dimethylaminopropyl)-N’-ethylcarbodiimide hydrochloride was purchased from Merck KGaA (25952-53-8). MWCNTs were obtained from Chengdu Organic Chemicals Co., Ltd., Chengdu, China, with a diameter ranging from 20 to 30 nm and an average length of 50 μm. The main component of MWCNTs is carbon, which forms the skeleton of their nanotubes. Because of their excellent electrical conductivity and mechanical strength, MWCNTs are widely used in many high-performance materials. The MWCNTs used have a high purity of greater than 95%, where less than 3% amorphous carbon affects electrical conductivity and strength, and less than 1.5% ash refers to the catalyst residues used in the synthesis process. Prior to use, MWCNTs were processed and physio-chemically characterized as previously described [31,32,33,34,35,36,37,38,39,40,41,42,43,44,45,46,47].

In brief, MWCNTs were oxidized in a concentrated H_2_SO_4_/HNO_3_ mixture (3:1 by volume) for 12 h, followed by probe sonication at 750 watts for 100 s. The oxidized products were subsequently rinsed and filtered using a Millipore^®^ membrane (pore size: 2 μm, Burlington, MA, USA) and distilled water (18.2 Ω) until the pH returned to baseline, followed by drying in a vacuum oven at 50 °C. A MWCNT solution (2 mg/mL) was prepared by thoroughly drying the MWCNTs at 50 °C in a vacuum oven, dispersing them in distilled water via sonication, further dispersing with probe sonication at 360 watts for 60 s using an ultrasonic instrument, and then centrifuging at 1540× *g*/min for 20 min to remove undispersed substances from the aqueous phase.

To improve the dispersity of MWCNTs in a solution, the PEG needs to be evaluated. We considered MWCNT-PEG amounts of 0 g/mL, 10 g/mL, and 25 μg/mL, respectively. Too little PEG may not be effective in improving dispersion, while too much PEG may lead to aggregation. In biomedical applications, the amount of PEG needs to be optimized to ensure its biocompatibility and reduce cytotoxicity. PEG-modified MWCNTs were prepared according to previously reported methods [37]. Briefly, a mixture of 1 mg/mL MWCNT dispersion and 10 mg/mL PEG polymers underwent 30 min of bath sonication in the presence of 3 mg/mL N-(3-dimethylaminopropyl)-N’-ethylcarbodiimide hydrochloride. The reaction mixture was stirred at 24 °C for 12 h, and excess PEG polymers were removed by filtration through Amicon Ultra centrifugal filters with a molecular weight cutoff of 100 kDa (Millipore, Carrigtwohill, Ireland), followed by five washes with water. Concentrations of MWCNTs and MWCNTs-PEG were determined using an ultraviolet spectrophotometer (DU800, Beckman, Brea, CA, USA) by measuring absorbance at 260 nm.

### 2.2. Scanning Electron Microscopy (SEM)

The solutions of MWCNTs and MWCNT-PEG were applied onto a glass substrate and air-dried at 24 °C for observation under SEM (S-4700, Hitachi, Tokyo, Japan). The length distribution of oxidized MWCNTs was determined by randomly counting more than 400 nanotubes across 10 SEM images.

### 2.3. Cell Culture and Mice

The RAW264.7 mouse macrophage cell line and NIH3T3 cells infected with the MOL4070LTR virus were cultured in Dulbecco’s modified Eagle medium (DMEM) (HyClone, Logan, UT, USA) supplemented with 10% fetal bovine serum and 1% penicillin/streptomycin. They were both obtained from ATCC (Manassas, VA, USA). Cell cultures were maintained in a humidified cell culture incubator at 37 °C with 5% CO_2_/95% air. Only cells in the logarithmic growth phase with greater than 95% viability were used. For experiments involving ROS, Iscove’s modified Dulbecco’s medium (IMDM) (HyClone) replaced Roswell Park Memorial Institute RPMI-1640 medium (Gibco, Billings, MT, USA).

FVB/N and severe combined immunodeficiency (SCID) mice were purchased from Nanjing Biomedical Research Institute of Nanjing University (Nanjing, China). The mice were housed, bred, and handled under specific pathogen-free conditions. We used newborn mice, and the characteristics of the mouse were as follows: skin was bright red, the body was hairless, eyes were not yet open, both ears were adhered to the skin, and the weight was about 1.5 g. We collected blood from the retro-orbital sinus in mice [48]. All experimental procedures were conducted in compliance with the Laboratory Animal Management Regulations of the People’s Republic of China and the Laboratory Animal Management Policies of Nanjing Medical University.

### 2.4. Murine Leukemia Virus (MuLV)-Induced Leukemia Mouse Model

**Figure 1:**
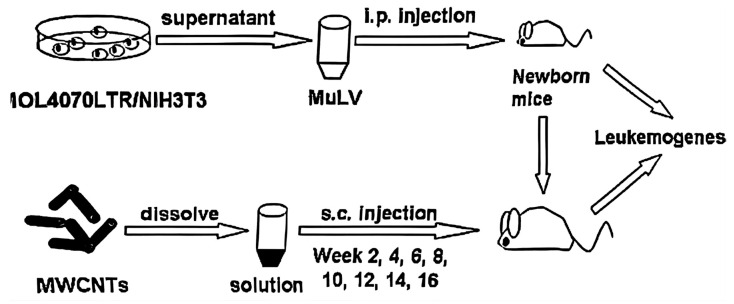
Schematic illustration depicting the procedure for establishing the MuLV-induced mouse model of leukemia.

We used a previously established mouse model of leukemia induced by the MOL4070LTR MuLV (Figure 1) [49]. MOL4070LTR viruses were prepared from infected NIH3T3 cells, generously provided by Dr. Linda Wolff of the US National Institutes of Health, and administered intraperitoneally to newborn mice [49]. Subsequently, 0.2 mg of MWCNTs or MWCNTs-PEG in 100 μL sterile water was injected subcutaneously every 2 weeks for the specified duration, starting 2 weeks after the initial injection of MOL4070LTR. Throughout the experiment, mice were monitored for signs of illness; those reaching a moribund state were euthanized by CO_2_ gas and subjected to analysis.

### 2.5. Detection of Pro-Inflammatory Cytokines by Enzyme-Linked Immunosorbent Assay (ELISA)

Serum levels of the pro-inflammatory cytokines IL-1β, IL-6, and TNF-α were quantified using Multiplex ELISA Kit following the manufacturer’s protocol (Boster Biological Technology, Wuhan, China, Catalog#MEK1011). Data were acquired and analyzed using a 96-well plate reader (SpectraMax M5, Molecular Devices, Sunnyvale, CA, USA) equipped with the software SoftMax® Pro-5 (Molecular Devices). Standard curves were generated based on the optical density (OD450) values of standards at various concentrations, yielding coefficients of determination (R2) of 0.98, 0.99, and 0.98 for IL-1β, IL-6, and TNF-α, respectively. Cytokine concentrations were calculated using standard curves.

### 2.6. Histopathology

Blood smears were stained with Wright-Giemsa. Mouse tissues were fixed in 4% paraformaldehyde, embedded in paraffin, sectioned, and stained with hematoxylin and eosin. Microscopic images of blood smears and tissue sections were captured using a microscope (FSX100, Olympus, Tokyo, Japan). Additionally, postmortem measurements of liver and spleen weights were recorded.

### 2.7. Uptake of MWCNTs and MWCNTs-PEG by Transmission Electron Microscopy (TEM) and Concentration Determinations

RAW264.7 cells were exposed to 50 μg/mL of MWCNTs or MWCNTs-PEG, or phosphate-buffered saline (PBS) as a control, for 72 h. Supernatants were collected and analyzed for MWCNT or MWCNT-PEG concentrations using ultraviolet spectroscopy to assess cellular uptake by RAW264.7 cells. Subsequently, cells were rinsed with 1 × PBS, fixed with 2.5% glutaraldehyde at 4 °C overnight, washed again, and then fixed with 1% aqueous osmium tetroxide. Following fixation, cells were embedded in araldite resin and sectioned into 80 nm ultra-thin slices for examination using a TEM (HT7700, Hitachi, Tokyo, Japan). Images were captured during the observation process.

### 2.8. Flow Cytometry Analysis of Mouse Leukemic Cell Lineage

Mouse leukemia cells, isolated from bone marrow (BM) and lymph nodes (LNs), were incubated with fetal calf serum to block nonspecific binding before incubation with fluorochrome-conjugated antibodies (Biolegend, San Diego, CA, USA). To identify myelogenous leukemia, cells were doubled stained with fluorescein isocyanate (FITC)-conjugated anti-mouse CD11b antibody (Biolegend, 11-0112) and PE-conjugated anti-mouse Gr-1 antibody (Biolegend, 108407). To identify lymphoid leukemia, cells were doubled stained with PE-conjugated anti-mouse TCR-β antibody (Biolegend, 984702) and fluorescein isocyanate (FITC)-conjugated anti-mouse B220 antibody (Biolegend, 103205). Antibody reagents were added separately following the manufacturer’s protocol, and then strained at 4 °C for 15 min in the dark. After staining, cells were washed with PBS buffer twice and then resuspended in PBS buffer. The cells were analyzed using a fluorescence activated cell sorting (FACS) calibur (BD Biosciences, Franklin Lakes, NJ, USA) with FlowJo^®^ software (version 9.5). This immunophenotyping procedure was routinely performed in our laboratory for characterizing mouse leukemia cells [48].

### 2.9. ROS Detection

Intracellular levels of ROS were measured using a Reactive Oxygen Species Assay Kit (Beyotime Biotechnology, Shanghai, China, Catalog#S0033M) according to the manufacturer’s instructions. Briefly, RAW264.7 cells were harvested by centrifugation at 600× *g* for 5 min, resuspended in a 10 μM solution of 2’,7’-Dichlorofluorescin diacetate (DCFH-DA) to achieve a final density varying form 1 × 10^6^ to 10^7^ cells/mL, and then incubated at 37 °C for 20 min. After incubation, cells were washed with serum-free medium to remove extracellular DCFH-DA, and ROS levels were quantified using a FACS Calibur flow cytometer with FlowJo^®^ software (version 9.5).

### 2.10. Statistical Analyses

Data were analyzed using SPSS 11.0 statistical software and presented as mean ± standard deviation (SD) from at least three independent experiments. Differences between two independent variables were compared using two-tailed Student’s *t*-tests, and we used ANOVA as a multiple comparison test, with *p*-values < 0.05 considered significant. Kaplan–Meier estimates were used to generate survival curves. Given the uncertainty of the exact onset of leukemia in mice and the observation of widely disseminated leukemia at sacrifice and autopsy, the interval from MuLV injection to sacrifice was used as a surrogate for leukemia development rate and to compute cumulative leukemia incidence. The log-rank test was used to calculate *p*-values between groups.

## 3. Results

### 3.1. Characterization of MWCNTs

**Figure 2:**
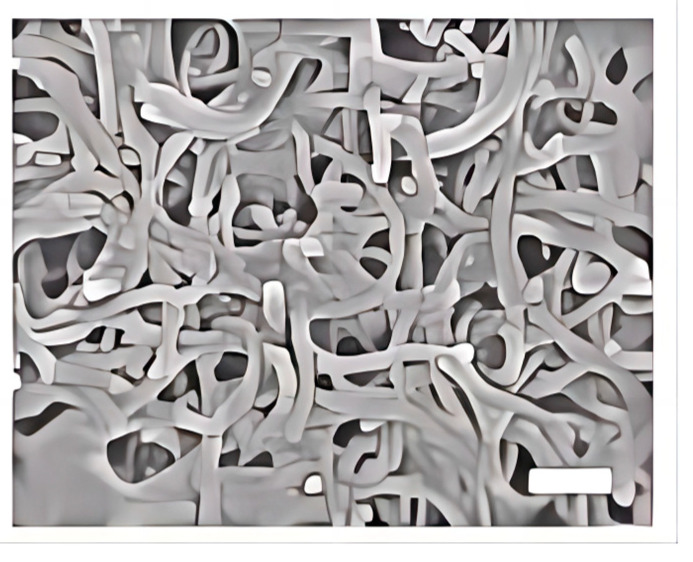
Tubular structure of oxidized MWCNTs. (SEM image showing MWCNTs used in the study, scale bar: 200 nm).

Considering that CNT length was a critical parameter influencing biological effects, we used SEM to characterize the morphology and the size of oxidized MWCNTs. SEM imaging revealed typical tube-like structures for oxidized MWCNTs (Figure 2). A length distribution analysis of oxidized MWCNTs involved the statistical counting and measurement of more than 400 nanotubes randomly selected from 10 SEM images. The size distribution ranged from 500 nm to 2 μm, with an average length of 970 nm. The MWCNT solution was well dispersed and relatively homogeneous. The Zeta potential value when dispersed in water was measured by Zetasizer 3000HS at −47.9 mV.

### 3.2. Mouse Model Data

**Figure 3:**
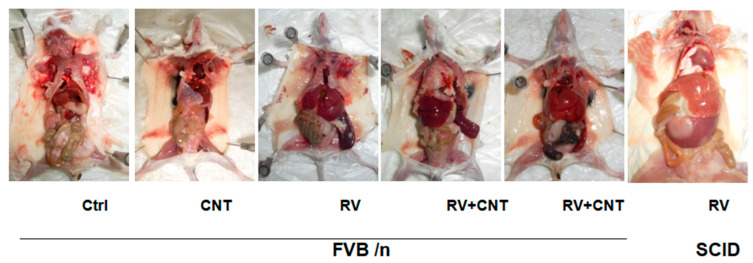
Gross anatomy of MuLV-induced leukemia in mice. (Ctrl is the control; CNT: MWCNTs; RV: MuLV; SCID and FVB/n are both mouse strains. RV + CNT is the combination of MuLV and MWCNTs).

Due to potential stress from multiple injections, each group of mice received identical numbers of injections of MuLV, MWCNTs, vehicle (1 × PBS), or combinations at consistent time points within each experiment, by injecting different drugs into mice and dissecting the mice after death (Figure 3).

**Figure 4:**
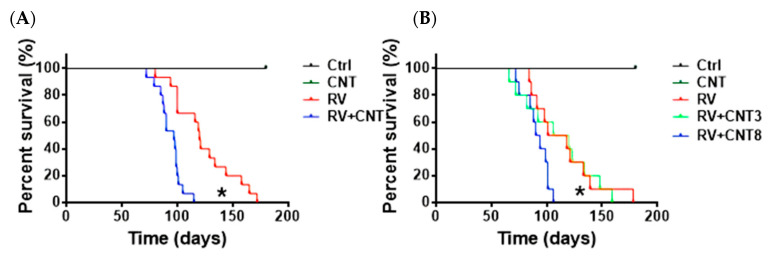
Survival probability of mice injected with MuLV alone or in combination with MWCNTs. (**A**). Cumulative incidence of leukemia in mice injected with PBS (Control; n = 15), MuLV (n = 15), 8 injections of MWCNTs (n = 15), or MuLV + 8 injections of MWCNTs (n = 15) (* MuLV vs. MuLV + MWCNTs; *p* = 0.01); (**B**). Cumulative incidence of leukemia in mice injected with PBS (Control; n = 10), MuLV (n = 10), MWCNTs (n = 10), MuLV + 3 injections of MWCNTs (n = 10), or 8 injections of MWCNTs (n = 10). (* MuLV + MWCNTs 8 vs. MuLV + MWCNTs 3: *p* = 0.03).

Mice injected with MuLV alone or in combination with MWCNTs began displaying abnormalities approximately 80 days following injection, including weight loss, elevated white blood cell counts, and reduced physical activity. All injected mice became moribund and exhibited leukemia at sacrifice. In the group receiving combined injections (including eight MWCNT injections), mice reached a median survival of 97 days (95% confidence Interval (CI): 72, 122 days), compared to 118 days (95% CI: 93, 143 days; *p* = 0.01) for the MuLV control cohort (Figure 4A). Continued MWCNT injections caused hardening and darkening of the skin at the injection site, with observed MWCNT accumulation in the subcutaneous tissue compared to controls [11]. There was a correlation between MWCNT exposure and the rate of leukemia acceleration. Mice exposed to three MWCNT injections had a median time to leukemia of 100 days (95% CI: 69, 131 days), whereas those receiving eight injections had a median of 91 days (95% CI: 60, 122 days; *p* = 0.03), and MuLV-only mice had a median of 117 days (95% CI: 87, 147 days; *p* = 0.82; Figure 4B).

**Figure 5:**
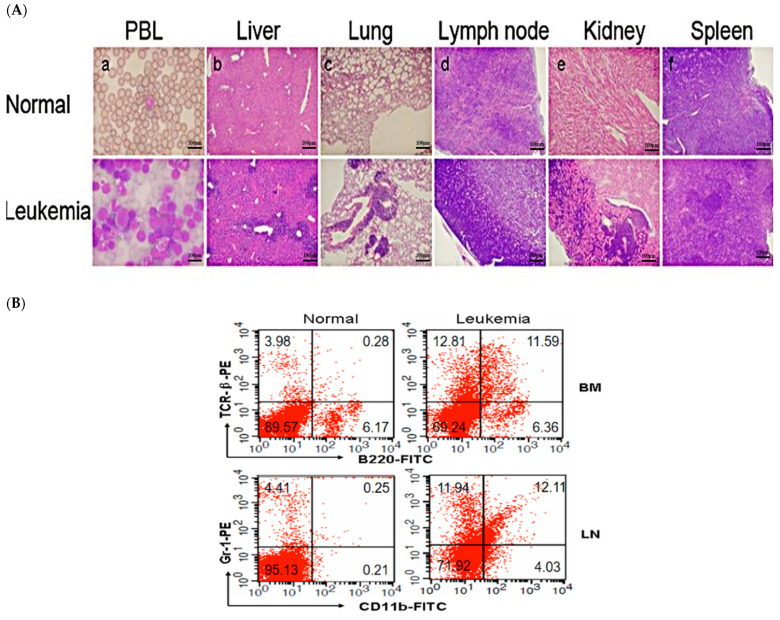
Effect of MWCNTs on MOL4070LTR-induced leukemia. (**A**). H&E-stained sections of (**a**) blood; (**b**) liver; (**c**) lung; (**d**) lymph node; (**e**) kidney; and (**f**) spleen from normal (upper panel) and leukemic (lower panel) mice. (**B**) Immunostaining of BM and LN cells from normal (left panel) or leukemic (right panel) mice with fluorescence-conjugated antibodies recognizing TCR-β+/B220+ (upper panel) or CD11b+/Gr-1+ (lower panel). Numbers in quadrants indicate percentage of each subpopulation. LN, lymph node; BM, bone marrow; H&E, hematoxylin and eosin).

**Figure 6:**
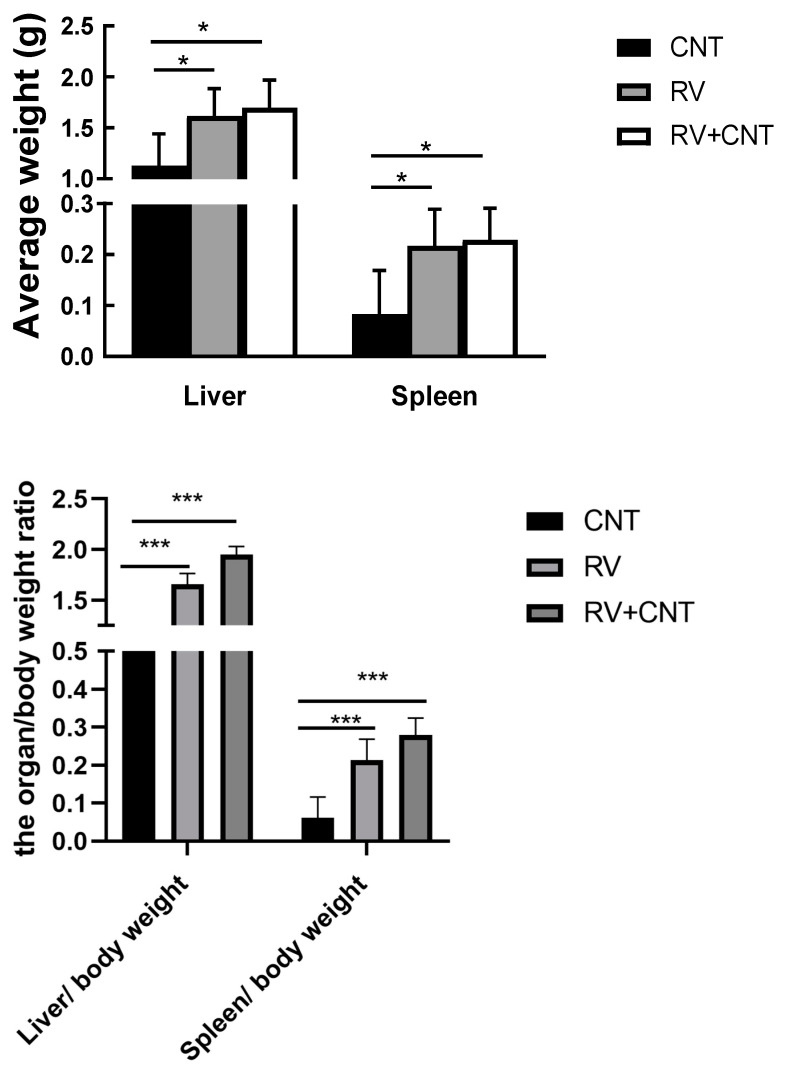
Weight map of spleen and liver of mice; the organ/body weight ratio after the test (spleen and liver weights; the organ/body weight ratio for mice receiving MWCNTs, MuLV, and MWCNTs + MuLV (* *p* < 0.05), (*** *p* < 0.001)).

Blood cells from mice with leukemia showed various features, such as open chromatin, loose chromatin structure, multiple nucleoli, and irregular nuclear contours (Figure 5A(a)). These mice also presented with enlarged livers, spleens, LNs, and occasionally thymi (Figure 6). Histopathological analysis revealed widespread blast infiltration across all studied organs (Figure 5A(b–f)), with no significant differences observed in these parameters between cohorts. Bone marrow and lymph node cells from mice with leukemia were analyzed for lineage (Figure 5B). Analysis of BM and LN cells from mice with leukemia indicated various leukemia lineages, including myeloid, lymphoid, and occasional bi-phenotypic leukemias (Table 1). Frequencies of leukemia types did not significantly differ among the cohorts (Table 2). At sacrifice, peripheral blood parameters of mice with leukemia diverged from those of control mice (Table 3).

### 3.3. Effect of PEG Modification of MWCNTs

**Figure 7:**
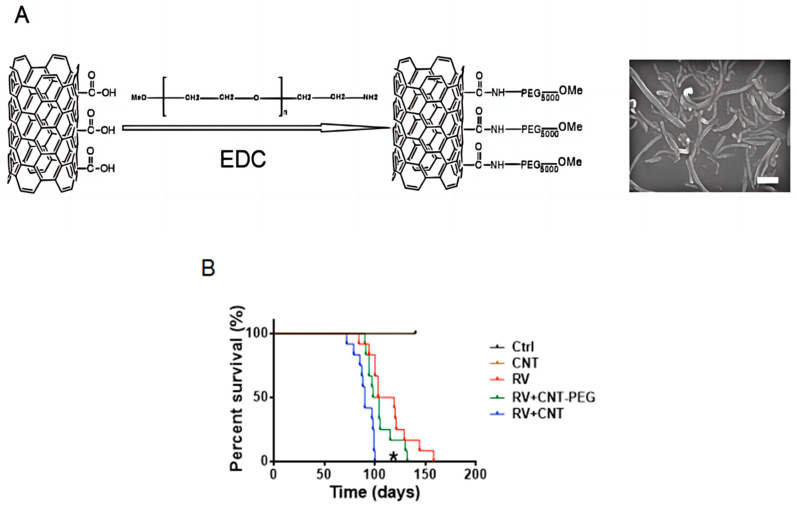
Effect of MWCNTs-PEG on MOL4070LTR-induced leukemia. (**A**) SEM image of MWCNTs-PEG (scale bar: 200 nm); (**B**). Cumulative incidence of leukemia in mice receiving PBS (Control; n = 12), MuLV only (MuLV; n = 12), MWCNTs alone (n = 12), MuLV + MWCNTs (n = 12), or MuLV + MWCNTs-PEG (n = 12). (* MuLV + MWCNTs vs. MuLV or MuLV + MWCNTs-PEG; both *p* = 0.01). MWCNTs, multi-walled carbon nanotubes; PEG, methoxypolyethylene glycol amine; PBS; phosphate-buffered saline.

SEM images showed MWCNTs-PEG with an average length of 970 nm (Figure 7A). However, the Zeta potential decreased significantly to −11.4 mV (*p* = 0.01). Median survival intervals for mice receiving unmodified or modified MWCNTs were 88 days (95% CI: 60, 116 days) and 100 days (95% CI: 72, 128 days; *p* = 0.01), respectively. No significant differences in leukemia types were found among the groups (Figure 7B).

### 3.4. Effects of MWCNTs in Immune Deficient Mice

**Figure 8:**
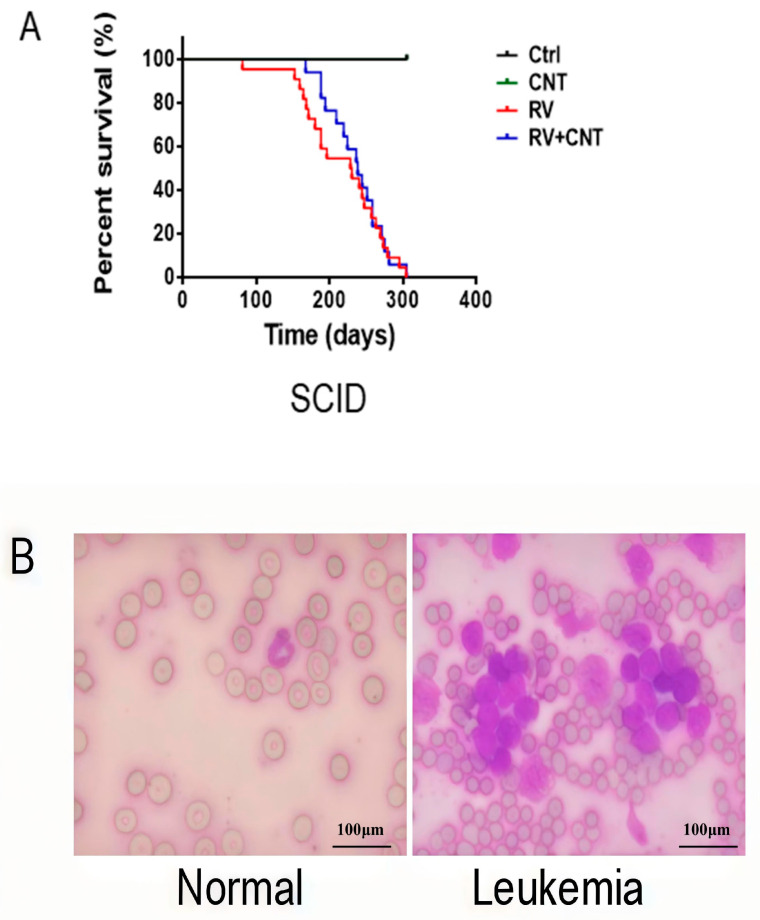
Effect of MWCNTs on leukemia development in immune deficient mice. (**A**) Cumulative incidence of leukemia in SCID mice receiving PBS (Control; n = 20), MuLV only (n = 22), MWCNTs only (n = 20), or MuLV + MWCNTs (n = 17); (**B**) Wright-Giemsa staining, representative blood smears of healthy (left panel) and immune deficient mice (right panel).

SCID mice were injected with MuLV either alone or in combination with three doses of MWCNTs. Mice injected with MuLV alone died from leukemia with a median survival of 236 days (95% CI: 215, 257 days), compared to 240 days (95% CI: 215, 265 days; *p* = 0.30; Figure 8A) for those with three injections of MWCNTs. Leukemia cells observed in both cohorts were similar (Figure 8B), as were the postmortem weights of liver, spleen, and LNs. Interestingly, the group of SCID mice that received the combination of MuLV and MWCNTs exhibited a slight, yet statistically insignificant, increase in median survival time. This suggests that the co-administration of MWCNTs may not significantly alter the progression of leukemia in these mice. However, further investigation into the potential effects of MWCNTs on the overall health and immune response of the mice is warranted.

### 3.5. Pro-Inflammatory Cytokines in Mice Exposed to MWCNTs

**Figure 9:**
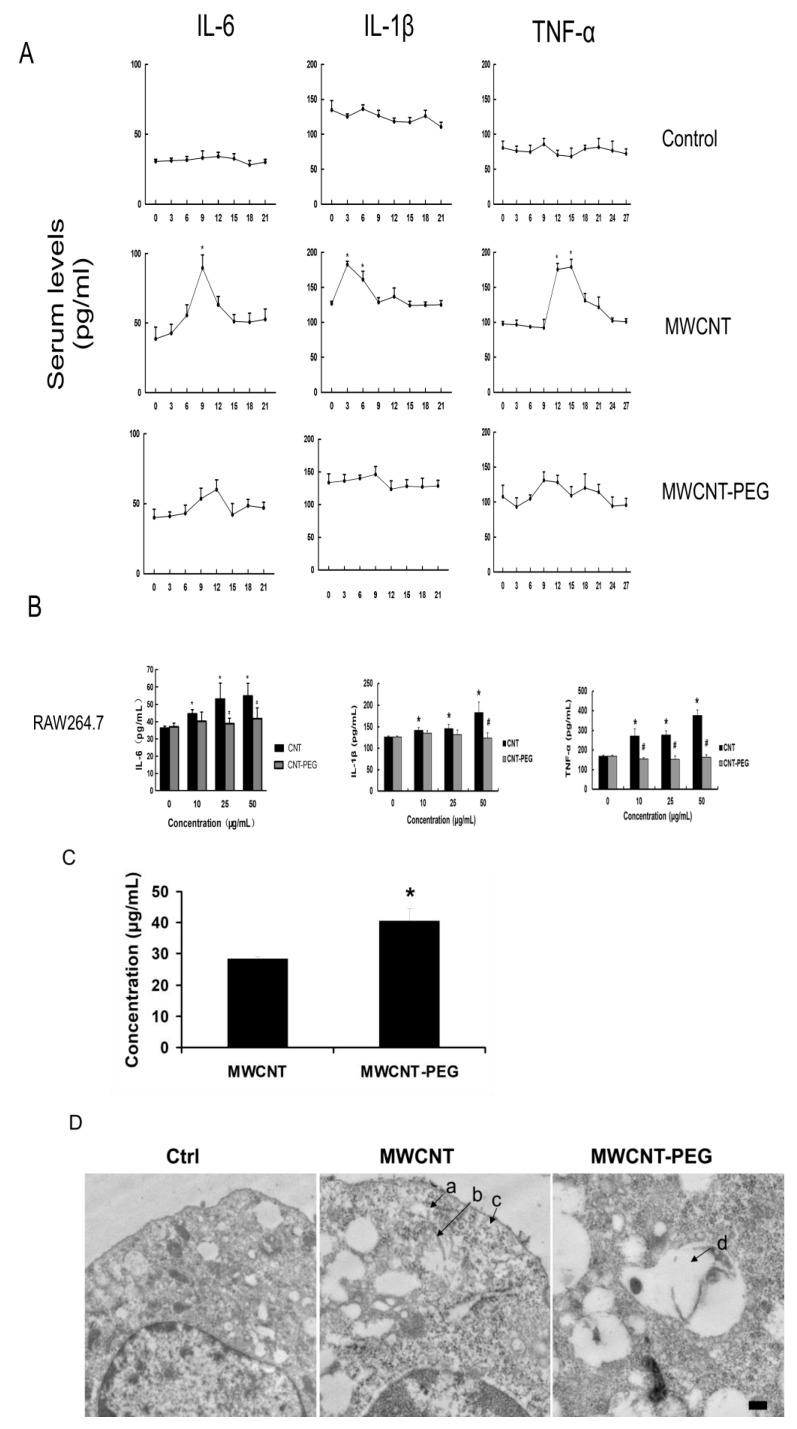
Pro-inflammatory cytokines in mice injected with MWCNTs or MWCNTs-PEG. (**A**) Serum levels of IL-6, IL-1β, and TNF-α. Mean ± SD of 8 mice per time point (*p* = 0.01); (**B**) concentrations of IL-6, TNF-α, and IL-1β in supernatants of mouse macrophage cell line RAW264.7 co-incubated with MWCNTs or MWCNTs-PEG at indicated concentrations for 48 h (*p* < 0.05; MWCNTs vs. Control; # *p* < 0.05; MWCNTs-PEG vs. MWCNTs at same concentration). (**C**) Supernatant concentrations of MWCNTs and MWCNTs-PEG measured by UV absorbance, as described in Methods. (* *p* = 0.01; MWCNTs vs. MWCNTs-PEG.) (**D**) Transmission electron microscopy (TEM) images showing the uptake of MWCNTs or MWCNTs-PEG by RAW264.7 cells. Scale bar: 200 nm. MWCNTs, multi-walled carbon nanotubes; PEG, methoxypolyethylene glycol amine; TEM, transmission electron microscopy.

Mice were injected with either MWCNTs or MWCNTs-PEG, and serum samples were collected every 3 days for 21 days to measure IL-6 and IL-1β levels, or for 27 days to measure TNF-α levels. Following MWCNT injection, IL-6 levels increased significantly from a baseline of 35.6 ± 7.7 pg/mL to 87.3 ± 11.1 pg/mL (*p* = 0.01) on day 9, subsequently decreasing to 48.7 ± 10.5 pg/mL by day 18. IL-1β levels increased from a baseline of 133.7 ± 7.1 pg/mL to 183.2 ± 10.2 pg/mL (*p* = 0.005) by day 3, and then returned to 131.8 ± 11.2 pg/mL by day 21 (*p* = 0.85). TNF-α levels increased from a baseline of 97.2 ± 8.1 pg/mL to 174.8 ± 9.7 pg/mL (*p* = 0.001) on day 12, peaked at 180.1 ± 8.1 pg/mL on day 15, and subsequently declined to 96.3 ± 10.4 pg/mL by day 27 (*p* = 0.92). In contrast, levels of IL-6, IL-1β, and TNF-α were not significantly altered by the injection of MWCNTs-PEG (Figure 9A).

### 3.6. Effects of MWCNTs on Macrophages

RAW264.7 cells were cultured with MWCNTs or MWCNTs-PEG at concentrations ranging from 0 to 50 μg/mL for 48 h, and supernatants were analyzed for IL-6, IL-1β, and TNF-α levels. Levels of all three cytokines were significantly higher in cells exposed to MWCNTs compared to those exposed to MWCNTs-PEG (Figure 9B). Additionally, we assessed the phagocytosis of MWCNTs and MWCNTs-PEG by RAW264.7 cells exposed to 50 μg/mL for 72 h. The concentration of MWCNTs in the supernatant decreased to 26.1 μg/mL compared to 40.96 μg/mL for MWCNTs-PEG (*p* < 0.01; Figure 9C). TEM images revealed the internalization of both MWCNTs and MWCNTs-PEG by RAW264.7 cells, with single nanotubes distributed in the cytoplasm (arrow a), clustered (arrows b and c), or contained within vacuoles (arrow d) (Figure 9D). RAW264.7 cells internalized significantly more MWCNTs (10 ± 2 nanotubes per cell) compared to MWCNTs-PEG (2 ± 1 nanotubes per cell; *p* < 0.01). Incubation with either MWCNTs or MWCNTs-PEG (0 to 50 μg/mL) for 48 h did not detectably affect the cell cycle or apoptosis, as analyzed by flow cytometry.

### 3.7. ROS Levels

**Figure 10:**
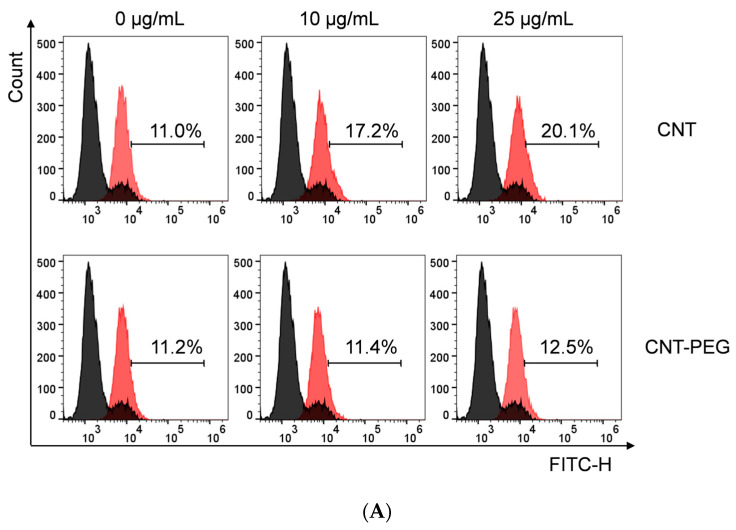

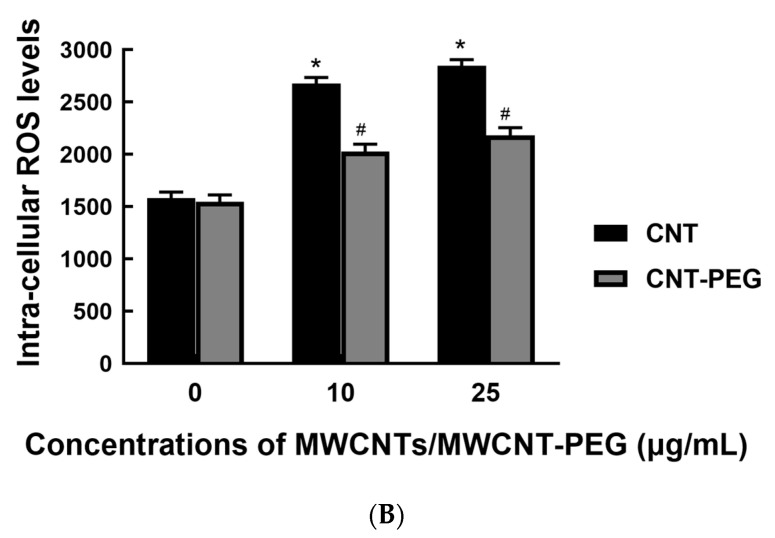
Effect of MWCNTs on intra-cellular ROS levels. (ROS levels in RAW264.7 cells following co-incubation with MWCNTs or MWCNTs-PEG at indicated concentrations for 16 h, prior to DCFH staining.) (**A**) Flow cytometry detection results of cellular ROS levels. (**B**) Quantitative analysis of cellular ROS levels in each group. * *p* = 0.001, # *p* < 0.05. *: MWCNTs vs. Control; #: MWCNTs-PEG vs. MWCNTs. MWCNTs, multi-walled carbon nanotubes; PEG, methoxypolyethylene glycol amine; ROS, reactive oxygen species; DCFH, 2’,7’-Dichlorofluorescin).

To assess the effects of MWCNTs and MWCNTs-PEG on intracellular ROS concentrations, RAW264.7 cells were exposed to MWCNTs and MWCNTs-PEG at 10 g/mL and 25 μg/mL for 16 h. Flow cytometry analysis revealed increased intracellular ROS levels following treatment with both MWCNTs and MWCNTs-PEG, although the increase was significantly lower with MWCNTs-PEG compared to MWCNTs (Figure 10).

## 4. Discussion

Although MWCNTs are increasingly used in medical applications, including drug formulation, their safety remains uncertain for individuals with hematologic cancers. Our study demonstrated that MWCNT injections accelerated leukemia development in mice latently infected with MuLV. This acceleration correlated with elevated blood levels of pro-inflammatory cytokines and increased intracellular ROS concentrations in macrophages. Notably, this effect was absent in immune-deficient mice and when the MWCNTs were surface-coated with PEG. These findings suggest that inflammation and immune modulation may play significant roles in accelerating latent leukemia development.

Previous studies have highlighted potential adverse effects associated with MWCNT exposure. For instance, intravenous administration of MWCNTs in pregnant mice disrupted fetal development [50]. Long-term exposure of human lung epithelial cells to single-walled CNTs induced neoplastic properties such as enhanced self-renewal, invasion, and tumorigenesis over six months [51]. Furthermore, intra-peritoneal injection of MWCNTs triggered mesothelioma in Trp53-deficient mice [52]. The size of MWCNTs might be important in these processes; smaller diameter tubes (approximately 50 nm) provoked inflammation and mesothelioma development, whereas larger diameter tubes (around 150 nm) exhibited reduced toxicity [19]. The particle size and the length of multi-walled carbon nanotubes (MWCNT) are closely and intricately linked to their biosafety profile. These physical characteristics play a crucial role in determining how these nanotubes interact with biological systems, and subsequently, their potential impact on health and the environment. The dimensions of MWCNT can influence their uptake by cells, their distribution within tissues, and their elimination from the body. Smaller particles and shorter lengths may facilitate easier clearance, reducing the likelihood of adverse effects, whereas larger and longer MWCNTs could pose greater risks due to their potential to cause physical damage or trigger immune responses. Understanding these relationships is essential for the safe design and application of MWCNT in various fields, including medicine. The particle size and the length of multi-walled carbon nanotubes (MWCNT) are closely and intricately linked to their biosafety profile [53,54].

In our study, the MWCNTs used had an average diameter of 20–30 nm and length of 50 μm. Results showed that nanoparticle dimensions and experimental model had a potential in determining the safety of CNT exposures. Importantly, we observed no typical carbon ash particles in TEM, indicating that the effects in this study could be attributable to MWCNTs rather than their reduction products.

We observed that administering eight bi-weekly injections of MWCNTs accelerated leukemia development in our experimental model. Given that all mice eventually developed leukemia, it appears that the rate of leukemia progression, rather than its occurrence, was affected. This acceleration could result from a quicker transition from a preleukaemic to leukemic state, enhanced growth of leukemia cells, or both mechanisms, which could not be differentiated in our model. Types of leukemias, their morphology, and organ distributions were comparable between mice with accelerated leukemia development and those without.

The mechanisms through which MWCNTs accelerate leukemia development remain unclear. We observed that MWCNT injections increased levels of pro-inflammatory cytokines in the blood and intra-cellular ROS in macrophages. Whether these changes are merely associations or causally linked remains uncertain. However, the absence of leukemia acceleration in immune deficient mice and in mice injected with MWCNTs-PEG supports a potential causal relationship. Consistent with this hypothesis, previous reports have indicated that MWCNTs stimulate macrophages to produce ROS and IL-1β, and activate fibroblasts, leading to myofibroblast transformation [21]. Recent studies have also implicated TNFα, IFNs, IL-1, and IL-6 in MPNs, MDS, and AML [22,23,24,25,26,27,28,29,30]. Furthermore, autoimmune disorders such as rheumatoid arthritis and lupus were associated with an increased risk of myeloid cancers [55,56]. Activation of inflammatory cytokine responses has been reported to drive the development of preleukaemic phenotypes in myelodysplastic syndromes in mouse models [54,55,56,57]. Mutations activating Ptpn11 in mesenchymal stem/progenitor cells and osteoid progenitors induced MPN in mice, possibly through excessive production of IL-1β and other pro-inflammatory cytokines by monocytes, hyper-activating hematopoietic stem/progenitor cells [58]. Additionally, loss of Dnmt3a in hematopoietic stem/progenitor cells in conjunction with Jak2V617F mutation led to lethal myelofibrosis by promoting activated inflammatory signaling [59,60,61,62,63,64,65].

The finding that immune deficient mice control leukemia development much later than the FVB/N strain suggests an interplay between leukemia development rate and immune function. It raises the possibility of an immune-stimulatory effect of MWCNTs rather than purely inflammatory. However, this hypothesis seems improbable. Moreover, using mortality as a surrogate for rate of leukemia development, we cannot exclude the possibility that MWCNT injections make mice with MuLV-induced leukemia more susceptible to death, although this scenario also appears unlikely.

## 5. Conclusions

**Figure 11:**
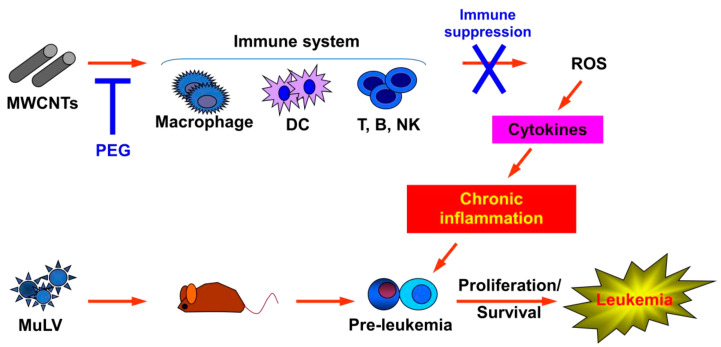
Model of potential mechanism of how MWCNTs accelerated leukemia development. MOL4070LTR induced leukemia in all susceptible mice. Injection of MWCNTs but not MWCNTs-PEG accelerated development by increasing ROS in macrophages and blood levels of pro-inflammatory cytokines. ROS, reactive oxygen species; MWCNTs, multi-walled carbon nanotubes; PEG, methoxypolyethylene glycol amine.

In summary, our study demonstrated that repeated injections of MWCNTs accelerated leukemia development in mice with latent MuLV infection, but not with immuno-compromised mice or MWCNT-PEG, suggesting a causal relationship between the acceleration and immune reaction (Figure 11). Viruses cause several human cancers, including certain lymphomas (Epstein-Barr virus), cervical and head and neck cancers (Human Papillomavirus), and Kaposi sarcoma (KSHV) [66]. Given potential exposure of individuals with these cancers to CNTs, comprehensive safety assessments in animal models and humans are crucial. Ongoing research involves investigating CNTs of varying sizes and using diverse experimental models. Further research should focus on elucidating the precise mechanisms by which MWCNTs interact with the immune system and viral pathogens to accelerate oncogenesis. Understanding these pathways could lead to the development of preventive strategies and targeted therapies for individuals at risk. Additionally, the impact of MWCNTs on other types of latent viral infections and their potential to induce other forms of cancer should be thoroughly investigated.

## Figures and Tables

**Table 1:** List of organs sampled.

	Organ
Immune System	Spleen
	Lymph Nodes
Respiratory System	Lung
Urinary System	Kidney
Digestive System	Liver

**Table 2:** Leukemia lineages.

Treatment	Myeloid	T	B	Mixed
MuLV (N = 15)	5	5	1	4
MuLV + MWCNTs (N = 15)	4	6	0	5

(Immune phenotyping by flow cytometry with antibodies to B220, TCR-β, CD11b and Gr-1.)

**Table 3:** Peripheral blood parameters.

Group of Mouse	WBC (×10^3^/μL)	Hemoglobin (g/L)	RBC (×10^6^/μL)	Platelet (×10^9^/μL)
Control	4.78 (0.46)	13.93 (0.25)	9.40 (1.01)	895.70 (36.92)
MuLV	6.18 (1.07)	13.48 (1.06)	8.24 (0.67)	841.43 (164.8)
MuLV + MWCNT	7.68 (2.02)	13.10 (0.78)	8.48 (0.64)	923.24 (176.68)
MuLV + MWCNT-PEG	6.73 (1.22)	12.36 (0.78)	8.43 (1.08)	915.03 (177.92)

(Values in brackets indicate standard derivation).

## Data Availability

All data generated or analyzed in the course of this study are included in this published article. If you are unclear, please ask the corresponding author.
