# Correction: Wang et al. Multi-Walled Carbon Nanotubes Accelerate Leukaemia Development in a Mouse Model. *Toxics* 2024, *12*, 646

**DOI:** 10.3390/toxics12110823

**Published:** 2024-11-18

**Authors:** Qingqing Wang, Jingdan Han, Mujia Wei, Huikai Miao, Min Zhang, Biao Wu, Yao Chen, Yanwen Zheng, Robert Peter Gale, Bin Yin

**Affiliations:** 1 Clinical Medical Research Center, The Affiliated Wuxi No.2 People’s Hospital of Nanjing Medical University, Wuxi 214002, China; wqq5837@163.com (Q.W.); hjd1356620009@163.com (J.H.); weimujiayouxiang@163.com (M.W.); 2 Department of Laboratory Medicine, Jiangnan University Medical Center, Wuxi 214002, China; miaohuikai1989@163.com (H.M.); mien1030@163.com (M.Z.); wubiao2011@163.com (B.W.); 15861681813@163.com (Y.C.); 3 Cyrus Tang Hematology Center, Jiangsu Institute of Hematology, The First Affiliated Hospital, Soochow University, Suzhou 215123, China; 13914391068@163.com; 4 Haematology Research Centre, Department of Immunology and Inflammation, Imperial College London, London SW7 2AZ, UK; robertpetergale@gmail.com

## 1. Author Removal

At the request of the authors, Haiyan Xu has been removed from the original publication [1].

## 2. Affiliation Update

In the publication [1], there was an error regarding the affiliations of Jingdan Han and Mujia Wei. The correct affiliations are listed below:

^1^ Clinical Medical Research Center, The Affiliated Wuxi No.2 People’s Hospital of Nanjing Medical University, Wuxi 214002, China

The authorship list of this publication has been amended to reflect this, and the Author Contributions statement has been updated as follows: 

**Author Contributions:** Conceptualization, Q.W. and J.H.; data curation, M.W.; formal analysis, H.M. and M.Z.; methodology, B.W. and Y.C.; writing—original draft, Y.Z.; writing—review and editing, R.P.G. and B.Y. All authors have read and agreed to the published version of the manuscript.

The authors state that the scientific conclusions are unaffected. This correction was accepted by the Editor-in-Chief. The original publication has also been updated.
